# Using Family History Data to Improve the Power of Association Studies: Application to Cancer in UK Biobank

**DOI:** 10.1002/gepi.22609

**Published:** 2025-01-03

**Authors:** Naomi Wilcox, Jonathan P. Tyrer, Joe Dennis, Xin Yang, John R. B. Perry, Eugene J. Gardner, Douglas F. Easton

**Affiliations:** ^1^ Centre for Cancer Genetic Epidemiology, Department of Public Health and Primary Care University of Cambridge Cambridge UK; ^2^ Metabolic Research Laboratory, Wellcome‐MRC Institute of Metabolic Science University of Cambridge Cambridge UK; ^3^ MRC Epidemiology Unit, Wellcome‐MRC Institute of Metabolic Science University of Cambridge Cambridge UK; ^4^ Centre for Cancer Genetic Epidemiology, Department of Oncology University of Cambridge Cambridge UK

**Keywords:** cancer, exome sequencing, family history, power calculations, protein‐truncating variants, UK Biobank

## Abstract

In large cohort studies the number of unaffected individuals outnumbers the number of affected individuals, and the power can be low to detect associations for outcomes with low prevalence. We consider how including recorded family history in regression models increases the power to detect associations between genetic variants and disease risk. We show theoretically and using Monte‐Carlo simulations that including a family history of the disease, with a weighting of 0.5 compared with true cases, increases the power to detect associations. This is a powerful approach for detecting variants with moderate effects, but for larger effect sizes a weighting of > 0.5 can be more powerful. We illustrate this both for common variants and for exome sequencing data for over 400,000 individuals in UK Biobank to evaluate the association between the burden of protein‐truncating variants in genes and risk for four cancer types.

## 1 Introduction

Genome‐wide association studies (GWAS) have been highly successful in identifying common variants associated with disease. Increasingly, association studies are being extended to study rare variants using next‐generation sequencing methods. Variants strongly predisposing to disease tend to be maintained at low allele frequencies in the population due to purifying selection, meaning that the power to detect rare variants individually is low, even in very large datasets such as UK Biobank, and careful consideration must be given to analysis methods. A common approach to improve power is to aggregate rare variants, for example, within genes, using burden tests, with the rationale that similar variants in the same gene are likely to have similar effects. A valuable additional source of information, often collected in research studies, is the family history of a disease. Individuals with affected relatives are more likely to carry risk variants than those without a family history. This phenomenon has been used to design more efficient GWAS by selecting cases enriched for family history (Antoniou and Easton 2003) and the same argument applies to rare variant association studies. Here we consider how such family history information should be best incorporated in rare variant association analyses. This is particularly relevant in the analysis of data from large cohort studies, in which the number of unaffected individuals far outnumber the cases and the number of unaffected individuals with a family history may be significant. For example, for diseases that are largely restricted to one sex (e.g., breast or prostate cancer), incorporating family history information allows data for individuals of both sexes to be utilised. Utilising ‘proxy’ family history data may also be important in situations where genotypes on cases cannot be obtained (e.g., diseases with high fatality).

Liu, Erlich, and Pickrell (2017) developed a method to test for associations between SNPs and disease by including family history information in controls. They defined ‘proxy cases’ as controls with affected first‐degree relatives, and ‘true controls’ to be controls with no affected first‐degree relatives. They tested for association between carrier status and the three possible outcomes (case, ‘proxy case’ or ‘true control’) using a 3 × 2 *χ* ^2^ test with three outcomes. This test showed greater power to detect associations than a 2 × 2 *χ* ^2^ test comparing just cases and controls (Liu, Erlich, and Pickrell 2017). An alternative method, which included information for first‐degree relatives of cases as well as controls, was suggested by Hujoel et al. (2020); the Liability Threshold Family History (LT‐FH) method. This has greater power than the Liu, Erlich, and Pickrell (2017) approach (Hujoel et al. 2020) and has also been used in a rare variant burden test framework: the family history aggregation unit‐based test (FHAT). This approach requires the full genotype matrix for each gene to calculate the test statistic (Wang et al. 2022). Our method has a similar rationale, using kinship and first‐degree relatives, but uses logistic regression directly on the aggregated burden variable.

In our approach, we consider a logistic regression model to test for an association between disease and gene variant burden that incorporates information on the family history of both cases and controls. Our proposed method weights the disease status of first‐degree relatives by *k*. We consider specifically the case *k* = 1/2, which is theoretically optimal under the limiting conditions of a rare disease and small effect size (see Section 2). We illustrate the method using data from UK Biobank to show how it improves power to detect associations for several cancers.

## 2 Methods

### 2.1 Model Motivation

We first consider association tests for a common variant. Let p be the frequency of the risk allele, which we assume is associated with an increased relative risk $e^{\beta }$ per allele, where β is assumed to be small. Let ${\underline{D}}_{i}=(D_{i0},D_{i1},..D_{\mathrm{im}(i)})$ be the disease phenotypes (0, 1) of the j=1..m(i) individuals in family i=1..n, where individual *j* = 0 is the typed proband, and ${\underline{g}}_{i}=(g_{i0},g_{i1},..g_{\mathrm{im}(i)})$ be the corresponding genotypes with observations $g_{\mathrm{ij}}=\;$0, 1 or 2 alleles. $t_{\mathrm{ij}}$ is the age of individual *i* in family *j*. We show in Supporting Information S1: Methods that the score test for *H*_0_: *β* = 0 has the form:

$$U=\sum_{i}\left(\sum_{j=0}^{m}\left(D_{\mathrm{ij}}-\Lambda_{0}\left(t_{\mathrm{ij}}\right)\right)2\phi_{\mathrm{ij}0}\right)\left(g_{i0}-2p\right).$$

With variance under the null:

$$V=2p\left(1-p\right)N\text{var}\left(\sum_{j=0}^{m(.)}\left(D_{.j}-\Lambda_{0}\left(t_{.j}\right)\right)2\phi_{.j0}\right),$$

Here $\phi_{\mathrm{ijk}}$ is the kinship coefficient between individuals *j* and *k* in pedigree *i* and $\Lambda_{0}\left(t\right)$ is the cumulative disease hazard in the population to age *t*. *N* is the total number of individuals (cases and controls). Thus, the (locally) most powerful test, for small effect sizes *β*, generalises a simple case–control analysis by replacing the disease status with the disease number of affected individuals in the pedigree, weighted by their degree of relationship to the proband, minus the weighted sum of the predicted number of affected individuals based on population incidence rates, and regressing this against the genotypes.

In practice, full pedigree data are not typically available: it is more usual for summary variables indicating a positive family history (yes/no) or the number of affected relatives, typically just first‐degree relatives, to be provided. If we assume that the disease phenotype is relatively rare so that most individuals are unaffected, then the $\Lambda_{0}\left(t_{\mathrm{ik}}\right)$ terms can be ignored, and the test reduces to regressing the genotypes against $D_{i0}+\frac{1}{2}f_{i}$., that is, the disease status of the proband plus ½ the number of affected first‐degree relatives f. In the analyses considered here, the possibility of more than 1 affected relative is ignored so that $f_{i}\;$ = 0 or 1.

### 2.2 Gene Burden Tests

Single variant association tests are generally underpowered for rare variants; however, burden tests, in which variants within units, for example, a gene, are collapsed together can be more powerful if the variants have similar effect sizes (Lee et al. 2014). Here we consider the simplest type of burden test where genotypes are collapsed to a 0/1 variable based on whether samples carry a variant of a given class (e.g., PTVs or rare missense variants in a specific gene). That is, $G_{i}=1\;\mathrm{if}\sum_{j=1}^{q}g_{\mathrm{ij}}>0\mathrm{and}0\mathrm{if}\sum_{j=1}^{q}g_{\mathrm{ij}}=0\;$where $g_{\mathrm{ij}}$ = 0, 1, 2 is the number of minor alleles observed for sample *i* at variant *j*, and *q* is the number of variants in the gene.

To apply the approach described above, we fit logistic regression models in which the carrier status is the outcome variable, and the disease phenotype is a covariate. Thus, a standard logistic regression model for the case–control study would be of the form:

$$\mathrm{Model}1:\;\log\left(\frac{P(G=1)}{1-P(G=1)}\right)=\alpha +\beta_{1}\mathrm{Case}+\beta_{3}\mathrm{Sex}+\beta_{4}x_{4}\ldots$$

Case is a binary variable indicating whether an individual is a case or control, Sex is a binary variable for female or male, and $x_{4}\ldots$ represent other covariates, for example, genetic principal components (PCs). *G* is a binary variable summarising the genotype (*G* = 1 for a carrier, *G* = 0 for a noncarrier).

This formulation of the model is the reverse of the more usual one in which disease status is the dependent variable and genotype is a covariate. However, as noted by Breslow and Day (Breslow 1987), the OR calculated in two‐by‐two tables remains consistent regardless of which variable is considered the dependent variable. Thus the estimated value of $\beta_{1}$ in this model is also an estimate of the log(OR) that would be obtained considering the disease status as the dependent variable, with potentially minor differences due to adjustment for other covariates. The model is formulated this way to facilitate extensions to include family history more straightforwardly.

To incorporate family history, we could add the covariate FH:

$$\mathrm{Model}2:\;\log\left(\frac{P(G=1)}{1-P(G=1)}\right)=\alpha +\beta_{1}\mathrm{Case}+{\beta_{2}\mathrm{FH}+\beta }_{3}\mathrm{Sex}+\beta_{4}x_{4}\ldots$$

FH is a binary variable indicating whether the individual has an affected first‐degree relative or not. A test for an association is a test of the null hypothesis $H_{0}{:\beta }_{1}=\beta_{2}=0$, leading to a 2‐degree of freedom (df) test. The test proposed by Liu et al is a $\chi^{2}$ score test, without additional covariates, but a likelihood ratio or Wald test is equivalent.

However, motivated by the arguments above, we consider a modification in which the effect size associated with family history is a predetermined fixed multiple of the case‐control effect size, that is, $\beta_{2}={k\beta }_{1}.$

$$\mathrm{Model}3:\;\log\left(\frac{P(G=1)}{1-P(G=1)}\right)=\alpha +\beta_{1}(\mathrm{Case}+{\mathrm{kFH})+\beta }_{3}\mathrm{Sex}+\beta_{4}x_{4}\ldots$$

This leads to a 1‐df test $H_{0}{:\beta }_{1}=0$. As above, we postulate that *k* = 1/2 is likely to be a reasonable choice. Model 1 is equivalent to *k* = 0, and *k* = 1 is equivalent to treating a positive family history as equivalent to a case. When fitting Model 2, we also calculate *k*_T_ = $\beta_{2}/\beta_{1}$ as the ‘true’ value of *k*.

For the main burden association results, generated using Model 3, we test for association using the Wald *p*‐value associated with $\beta_{1}$. However, to compare the power of Models 1–3 we use the likelihood ratio tests, comparing with the null model:

$$\mathrm{Model null}:\;\log\left(\frac{P(G=1)}{1-P(G=1)}\right)=\alpha +\beta_{3}\mathrm{Sex}+\beta_{4}x_{4}\ldots$$

In the case of rare alleles conferring a moderate risk, so that β is no longer close to zero, it is less clear that Model 3 with *k* = 1/2 provides the most powerful test. As an alternative motivation, we note that for a rare risk allele conferring a relative risk $e^{\beta }$, the allele frequency in individuals with an affected first‐degree relative is $\sim 2p\frac{1}{2}\left(e^{\beta }+1\right)$, that is, approximately the mean of the frequency in cases and controls (Risch 1990). This corresponds to a model in which $e^{\beta_{2}}=\frac{1}{2}\left(e^{\beta_{1}}+1\right)$. When $\beta =\beta_{1}$ is small this reduces to $\beta_{2}=\frac{1}{2}\beta_{1}$, that is, *k* = 1/2 as proposed. However, for larger effect sizes this predicts $\beta_{2}>\frac{1}{2}\beta_{1}$; hence, *k* > 1/2 may provide a more powerful test in this scenario. We examine the power of alternative values of *k*.

We report results for genes significant at exome‐wide significance (*p* < 2.5 × 10^–6^) and also at *p* < 0.001 (representing a level that defines a manageable number of genes to be followed up in further replication studies).

For higher frequency variants (such as SNPs in GWAS), the genotype *G* has three levels (0, 1, and 2). The equivalent to models 1–3 are then adjacent‐categories models:

$$\mathrm{Model}1:\;\log\left(\frac{P\left(G=j+1\right)}{P(G=j)}\right)=\alpha_{j0}+\beta_{1}\mathrm{Case}+\beta_{3}\mathrm{Sex},$$

$$\mathrm{Model}2:\;\log\left(\frac{P(G=j+1)}{P(G=j)}\right)=\alpha_{j0}+\beta_{1}\mathrm{Case}+{\beta_{2}\mathrm{FH}+\beta }_{3}\mathrm{Sex},$$

$$\mathrm{Model}3:\;\log\left(\frac{P(G=j+1)}{P(G=j)}\right)=\alpha_{j0}+\beta_{1}(\mathrm{Case}+{\mathrm{kFH})+\beta }_{3}\mathrm{Sex}.$$

In these models, $\beta_{1}$ is the per‐allele OR for the disease associated with the variant, and $\beta_{2}$ is the per‐allele OR for a positive family history.

### 2.3 Theoretical Power and Effective Sample Size

We can also derive approximate expressions for the gain in power achievable by incorporating family history in this way, expressed in terms of the relative sample size relative to a case–control study with equal numbers of cases and controls. Suppose there are *N*_*0*_ controls, *N*_*01*_ with a family history and *N*_*00*_ without, and *N*_*1*_ cases, and N_11_ with a family history and *N*_*10*_ without.

**Table:** 

	Control	Case
FH = 0	*N*_*00*_	*N*_*10*_
FH = 1	*N*_*01*_	*N*_*11*_
	*N*_*0*_	*N*_*1*_

We let $D_{\mathrm{ij}}=0,1$ be the disease status of individual *j* in family *i*, as above. Here $d_{i}=D_{i0}$ is the disease status of the proband, and $f_{i}=0, 1$ according to whether the proband has a positive family history or not. Let *p* be the population allele frequency and *β* the per‐allele rate ratio.

In the simple case–control analysis, excluding family history, the standard test is of the form:

$$U=\sum_{i}\left(d_{i}-\overset{®}{d}\right)\left(g_{i}-\overset{®}{g}\right)=\sum_{i}d_{i\prime }g_{i\prime }$$

With variance: $V=N\text{var}\left(\underline{d\prime }\right)\text{var}(\underline{g\prime })$

Where $d_{i}^{\prime }=d_{i}-\overset{®}{d}$ and $g_{i}^{\prime }=g_{i}-\overset{®}{g}$ are normalised phenotypes and genotypes with mean 0, and $N=N_{0}+N_{1}$ is the total number of genotyped individuals This gives a Z‐score of the form:

$$Z=U/\sqrt{V},$$

where, under the alternative (see Supporting Information S1: Methods):

$$E\left(Z|\beta \right)=\beta \sqrt{2p\left(1-p\right)\frac{N_{0}N_{1}}{N_{1}+N_{0}}}$$

This is a standard formula for deriving the power of a case–control study, excluding family history. The effective sample size is, by definition, the sample size of a case–control study with equal numbers of cases and controls that would give the same power, leading to the usual formula:

$$N_{\mathrm{eff}1}=\frac{2N_{0}N_{1}}{N_{1}+N_{0}}={2\left(\frac{1}{N_{1}}+\frac{1}{N_{0}}\right)}^{-1}.$$

Now we consider the test for our models, which is instead based on:

$$U=\sum_{i}(u_{i}-\overset{®}{u})\left(g_{i}-\overset{®}{g}\right)=\sum_{i}u_{i\prime }g_{i\prime }$$

Where the phenotype $u_{i}=d_{i}+\frac{1}{2}f_{i}$, with family history weighted by ½.

We show that (Supporting Information S1: Methods):

$$E\left(Z|\beta \right)=\beta \sqrt{2p\left(1-p\right)N\text{var}\left(\underline{u^{\prime }}\right)}=\beta \sqrt{p\left(1-p\right)\frac{1}{2N}\left(N_{00}N_{01}+N_{01}N_{10}+N_{10}N_{11}+4(N_{00}N_{10}+N_{01}N_{11})+9N_{00}N_{11}\right)}.$$

Therefore, the effective sample size is:

$$N_{\mathrm{eff}2}=\frac{1}{2N}\left(N_{00}N_{01}+N_{01}N_{10}+N_{10}N_{11}+4(N_{00}N_{10}+N_{01}N_{11})+9N_{00}N_{11}\right).$$

In the absence of family history, that is, $N_{01}=N_{11}=0$, this reduces to $\frac{{2N}_{00}N_{10}}{N}=\frac{{2N}_{0}N_{1}}{N}$ as expected.

It is possible to also calculate an effective population size if instead, the approach of Liu, Erlich, and Pickrell (2017) of grouping cases and controls with a family history (‘proxy cases’) together, is used. Here the phenotype $y_{i}=\max(d_{i},f_{i})$ (Liu, Erlich, and Pickrell 2017).

In this case, the test statistic is of the form:

$$U=\sum_{i}(y_{i}-\bar{y})\left(g_{i}-\bar{g}\right)=\sum_{i}y_{i\prime }g_{i\prime .}$$

With variance:

$$V^{\prime }=N\text{var}\left(y_{i}\right)\text{var}\left(g\right).$$

We show that (Supporting Information S1: Methods):

$$E(Z|\beta )=\sqrt{\frac{{N_{00}(N_{01}+{2N}_{10}+3N_{11})}^{2}p\left(1-p\right)}{2N(N_{10}+N_{10}+N_{11})}}.$$

Therefore, the effective sample size is:

$$N_{\mathrm{eff}3}=\frac{{N_{00}(N_{01}+{2N}_{10}+3N_{11})}^{2}}{2N(N_{10}+N_{10}+N_{11})}.$$

We derive effective sample sizes in UK Biobank for breast, prostate, bowel and lung cancer using these different approaches.

### 2.4 UK Biobank

UK Biobank is a population‐based prospective cohort study of more than 500,000 individuals. More detailed information on UK Biobank is given elsewhere (Collins 2012; Sudlow et al. 2015). WES data for 450,000 samples were released in October 2021 and accessed via the UK Biobank DNA Nexus platform (Backman et al. 2021). QC metrics were applied to Variant Call Format (VCF) files as described by Gardner et al including genotype level filters for depth and genotype quality (Gardner et al. 2022). Other filters including samples with disagreement between genetically determined and self‐reported sex, excess relatives, and so on, were applied as described elsewhere (Wilcox et al. 2023). Samples of ancestry other than European were excluded. The final data set for analysis included 419,307 samples with 227,393 females and 191,914 males.

Cases for breast cancer, prostate cancer, bowel cancer and lung cancer were determined by linkage to national cancer registration data (NCRAS) and selecting the appropriate ICD 10 codes (Supporting Information S1: Table 1). For breast cancer, we also included self‐reported cancer. Both prevalent and incident cases were included. Only cancers that were an individual's first or second diagnosed cancer were included as cases. For each cancer, controls are individuals without that given cancer (either by linkage or self‐report). The numbers of cases for males and females for each cancer are provided in Table 1.

**Table 1:** The number of female and male cases and controls for each cancer using the ICD‐10 codes in Supporting Information S1: Table 1.

	Females		Males	
	Control	Case	Control	Case
Breast cancer	209,435	17,958	191,820	94
Prostate cancer	227,393	0	180,249	11,665
Bowel cancer	224,404	2,989	187,956	3,958
Lung cancer	225,641	1,752	190,009	1,905

The Ensembl Variant Effect Predictor (VEP) was used to annotate variants, including the 1000 genomes phase 3 allele frequency, sequence ontology variant consequences and exon/intron number (McLaren et al. 2016). Annotation files were used to identify PTVs and rare (allele frequency < 0.001 in both the 1000 genomes data set and the current data set) missense variants. PTVs in the last exon of each gene and the last 50 bp of the penultimate exon were excluded as these are generally predicted to escape Nonsense‐Mediated mRNA Decay (NMD).

UK Biobank imputed genetic data was also accessed to explore results for four known breast cancer GWAS SNPs (Easton et al. 2007; Fachal et al. 2020; Michailidou et al. 2017; Turnbull et al. 2010). Genome‐wide genotyping was performed using the UK Biobank Axiom Array, where approximately 850,000 variants were measured, and this was imputed to > 90 million variants using the Haplotype Reference Consortium and UK10K + 10000 Genome's reference panels. This data set contained information for 451,959 individuals (245,215 females). Detailed information on the QC is provided by Bycroft et al. (2018).

### 2.5 Monte Carlo Simulations

We used Monte Carlo simulations to compare the power of models 2 and 3. We simulated *N* = 5000 datasets under alternate hypotheses with ORs from 1.1 to 5.0 and *k* value 0.5, each of size *n* = 450,000. For breast cancer, we also considered different values of *k* (from 0.3 to 1.0). The generated proportions of males and females, cases/controls and positive and negative family history were the same as for cancer phenotypes in the UK Biobank (Table 2); this was done using random sampling from a binomial distribution for each variable. We then generated a binary carrier variable, indicating whether or not an individual carried a risk variant, based on predicted probabilities from the logistic regression with coefficients (α, $\beta_{1}$ = log(OR), $\beta_{2}=\text{klog}(\text{OR})$, $\beta_{3})$. This was repeated for each of the 5000 simulated datasets. α was set at log(0.001) (i.e., assuming a baseline aggregate PTV frequency within genes of 0.001), and $\beta_{3}$ was set at log(1.15) representing the log(OR) associated with sex based on the $\beta_{3}$ value from the *CHEK2* analysis. The power was calculated as the proportion of times the null hypothesis was correctly rejected for the models, that is, the proportion of *p*‐values less than 0.05 for nominal significance, or less than 2.5 × 10^–6^ for exome‐wide significance, based on the appropriate likelihood ratio test. We also used Monte Carlo simulations with *N* = 5000 and *n* = 450,000, and the parameters of the breast cancer data, to evaluate the type‐1 error rate by simulating data under the null hypothesis with OR = 1.0 (Supporting Information S1: Table 2). These showed that the type I errors were consistent with expectations.

**Table 2:** Proportions used to simulate datasets for MC power calculations for the four cancers.

	Females	Cases within females	Cases within males	FH = 1 within female controls	FH cases within female cases	FH cases within male controls	FH cases within male cases
Breast cancer	0.54	0.079	0.00049	0.11	0.18	0.10	0.16
Prostate cancer	0.54	0	0.061	0.081	0	0.077	0.15
Bowel cancer	0.54	0.013	0.021	0.11	0.15	0.12	0.18
Lung cancer	0.54	0.0077	0.0099	0.13	0.23	0.13	0.22

## 3 Results

### 3.1 Breast Cancer: Known Genes

The ORs and *p*‐values for the five ‘known’ genes, under the different models, are given in Table 3. The association was most significant for Model 3 (assuming k = 1/2) for truncating variants in *ATM*, *CHEK2* and *PALB2*, and also for rare missense variants in *CHEK2*. For *BRCA1* and *BRCA2*, the association tests were more significant under Model 2. It is notable that for these genes, which are associated with the highest risks, the estimated values of *k* were the largest (all greater than 0.5), as expected. However, only for *BRCA1* and *BRCA2* did estimating *k* improve the significance, showing the benefit of Model 3 for detecting associations with modest effect sizes.

**Table 3:** ORs from models 1–3 for breast cancer risk for PTVs in known risk genes. For model 2 the ‘true’ $k_{T}{=\beta }_{2}/\beta_{1}\;$is calculated, and model 3 uses k = 1/2. *p*‐values are from the LRT to the Null model.

		Model 1	Model 2		Model 3
		OR	OR	*k*_T_	OR
PTVs	*ATM*	2.23 (1.79, 2.79), *p* = 1.25 × 10^–10^	2.14 (1.71, 2.68), *p* = 9.87 × 10^–17^	0.66	2.32 (1.94, 2.76), *p* = 1.77 × 10^–17^
	*BRCA1*	9.09 (6.75, 12.2), *p* = 4.66 × 10^–38^	7.64 (5.67, 10.3), *p* = 4.61 × 10^–78^	0.78	11.3 (8.97, 14.3), *p* = 1.26 × 10^–75^
	*BRCA2*	6.12 (5.22, 7.19), p = 1.31 × 10^–83^	5.42 (4.61, 6.37), *p* = 1.32 × 10^–151^	0.72	6.79 (5.98, 7.72), *p* = 2.35 × 10^–148^
	*CHEK2*	2.45 (2.11, 2.84), *p* = 2.29 × 10^–26^	2.34 (2.02, 2.72), *p* = 1.73 × 10^–42^	0.61	2.49 (2.21, 2.81), *p* = 2.57 × 10^–43^
	*PALB2*	4.03 (3.21, 5.04), *p* = 1.73 × 10^–26^	3.69 (2.95, 4.63), *p* = 9.26 × 10^–46^	0.70	4.33 (3.62, 5.18), *p* = 8.33 × 10^–46^
Rare missense variants	*CHEK2*	1.46 (1.30, 1.64), *p* = 8.93 × 10^–10^	1.44 (1.28, 1.62), p = 1.21 × 10^–12^	0.48	1.43 (1.31, 1.57), p = 1.29 × 10^–13^

### 3.2 Breast Cancer GWAS SNPs

**Figure 1:**
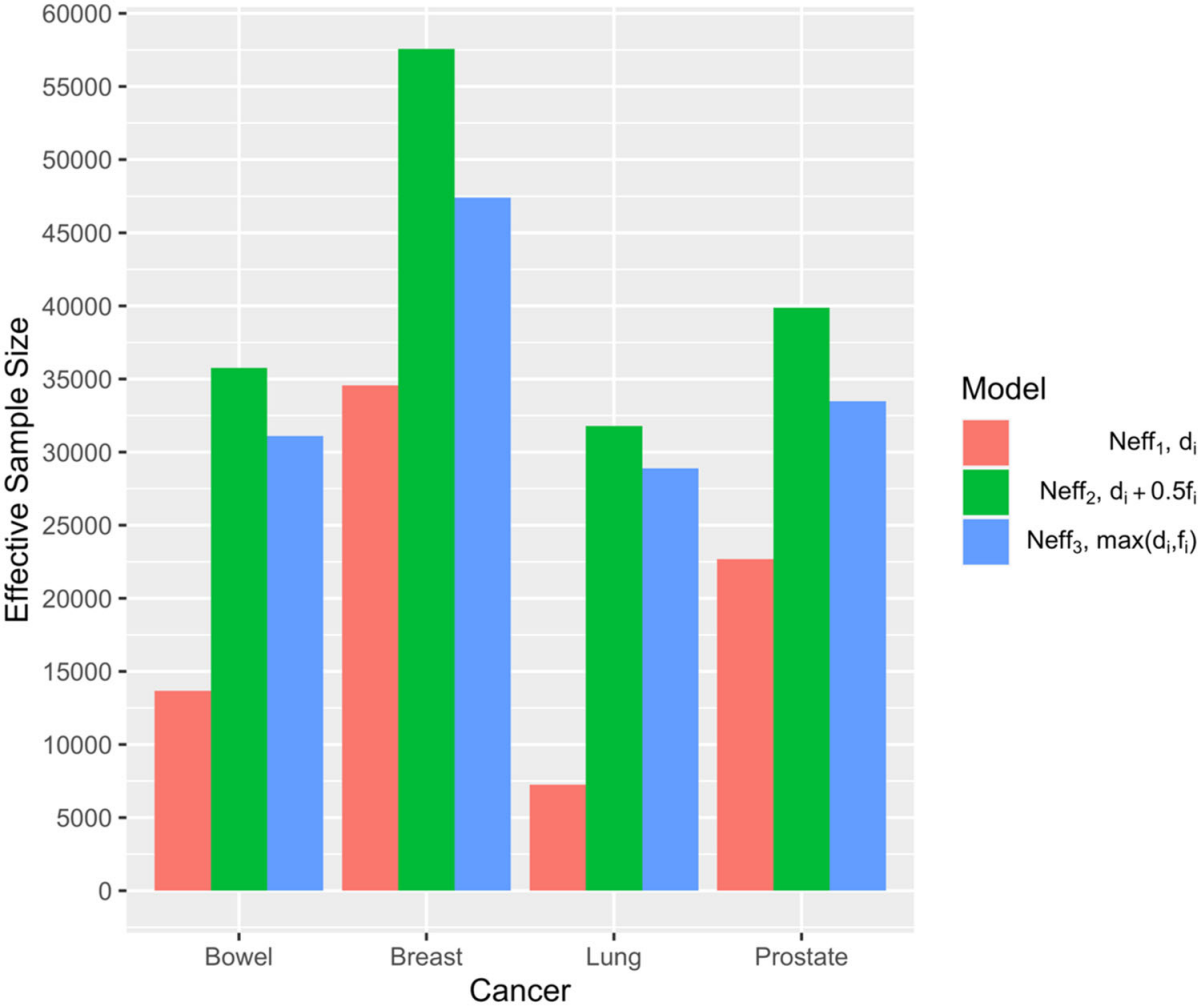
Effective sample size for different models. $N_{{\mathrm{eff}}_{1}}$ is the effective sample size from the standard case–control analysis (Model 1) just considering an individual's disease status, *d_i_*. $N_{{\mathrm{eff}}_{2}}$ is the effective sample size from our model including family history, $f_{i^{\prime }}$ of cases and controls with a weighting 0.5 (Model 3). $N_{{\mathrm{eff}}_{3}}$ is the effective sample size from the Model of Liu, Erlich, and Pickrell (2017) where controls with a positive family history are treated the same as true cases (Liu, Erlich, and Pickrell 2017).

Table 4 shows the corresponding results for GWAS SNPs. Here the analyses using model 3 (fixing *k* = 1/2) were consistently the most significant. Under Model 2, the best estimates of *k* are between 0.4 and 0.6, consistent with the theoretical expectation. In each case, the gain in efficiency (as measured by the ratio of the chi‐squared test statistics) for Model 3 versus Model is equivalent to an increase in sample size of ~1.5. This is close to the relative gain in theoretical effective sample size in Figure 1.

**Table 4:** Per‐allele ORs from Models 1–3 for 4 lead breast cancer GWAS SNPs. For Model 2 the ‘true’ $k_{T}{=\beta }_{2}/\beta_{1}\;$is calculated, and Model 3 uses *^k^* = 1/2. *p*‐values are from LRT to the null model.

SNP (gene)	Ref allele	Alt allele	ALT AF	Model 1	Model 2		Model 3
				OR	OR	*k*_T_	OR
rs62355901 *(C5orf67)*	T	C	0.162	1.17 (1.14, 1.21) *p* = 2.11 × 10^–30^	1.17 (1.14, 1.20) *p* = 7.07 × 10^–41^	0.43	1.16 (1.13, 1.18) *p* = 6.61 × 10^–42^
rs78540526 *(CCND1)*	C	T	0.070	1.31 (1.27, 1.36) *p* = 4.62 × 10^–45^	1.30 (1.25, 1.35) *p* = 3.44 × 10^–72^	0.56	1.31 (1.28, 1.35) *p* = 2.31 × 10^–73^
rs4784227 *(CASC16)*	C	T	0.240	1.26 (1.23, 1.29) *p* = 1.80 × 10^–83^	1.25 (1.22, 1.28) *p* = 4.09 × 10^–120^	0.46	1.24 (1.22, 1.27) *p* = 2.13 × 10^–121^
rs2981578 *(FGFR2)*	T	C	0.462	1.24 (1.22, 1.27) *p* = 1.45 × 10^–93^	1.24 (1.21, 1.26) *p* = 8.59 × 10^–131^	0.43	1.22 (1.20, 1.24) *p* = 1.24 × 10^–131^

### 3.3 Effective Sample Size

Figure 1 summarises the effective sample sizes for the analysis of the four cancer types in UK Biobank, using the different methods. As expected, the effective sample size is greatest for breast cancer, reflecting the higher prevalence of this cancer. The relative gain in effective sample size is, however, greatest for lung cancer (more than threefold) reflecting that the proportion of individuals with a positive family history is highest for this cancer.

### 3.4 Monte Carlo Simulations

We compared the power of Model 2 and Model 3 by Monte Carlo simulation for the four cancers.

**Figure 2:**
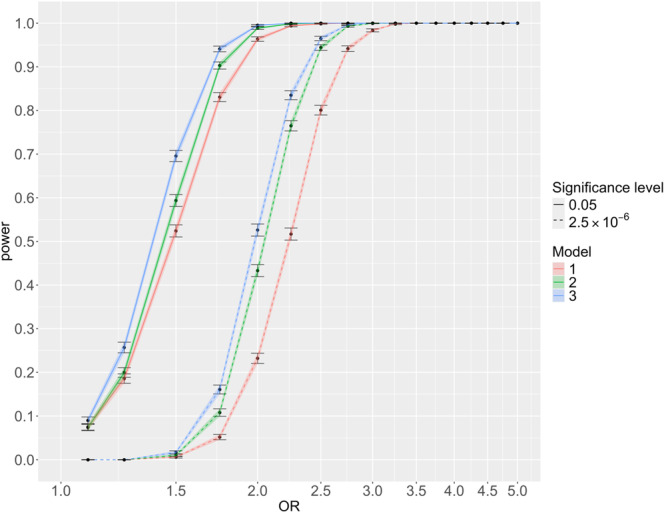
The power to detect different ORs for breast cancer risk. Power is calculated by Monte Carlo simulations for 5000 datasets of size 450,000 with proportions of sex, case/control, and family history the same as for breast cancer in UK Biobank. For each simulated data set Model 2 and Model 3 were compared to the null model by LRT. 95% confidence intervals for the power at each OR tested are shown.

For breast cancer, at a significance level of 0.05, the power is greater for Model 3 than Model 2 or Model 1 for OR < 2.5 (Figure 2). For example, the power to detect OR = 2 increases from 0.964 to 0.989 to 0.995 when comparing models 1 to 3. For OR ≥ 2.5 the power approaches 1 for all models. At exome‐wide significance, the power is also greatest for Model 3 for OR < 3. For example, the power to detect OR = 2 increases from 0.232 to 0.433 to 0.526 comparing Models 1–3. For OR ≥ 3 the power approaches 1 for all models. Varying the value of *k* between 0.4 and 0.7 under model 3 made little difference to the power for any OR (Supporting Information S1: Tables 4 and 5 and Figure 1).

For prostate cancer, the power of each model is lower than that for breast cancer (Supporting Information S1: Figures 2 and 3) reflecting the lower prevalence of the disease. At a significance level of 0.05, the power is greater for Model 3 than Model 2 or Model 1 for OR < 2.5 (Supporting Information S1: Figure 4). For example, the power to detect an association with OR = 2 increases from 0.86 to 0.94 to 0.97 comparing Models 1 to 3. For OR ≥ 2.5 the power approaches 1 for all models. At exome‐wide significance, the power is also greatest for Model 3 for OR < 3.5. For lung cancer, the power of each model is lower than that for breast, prostate, and bowel cancer (Supporting Information S1: Figures 2 and 3) reflecting the lower prevalence of the disease. At a significance level of 0.05, the power is greater for Model 3 than Model 2 or Model 1 for OR < 3 (Supporting Information S1: Figure 5). For example, the power to detect OR = 2 increases from 0.48 to 0.87 to 0.92 comparing Models 1–3. For OR ≥ 3.5 the power approaches 1 for all models. At exome‐wide significance, the power is also greatest for Model 3 for OR < 4. Finally, for bowel cancer, the power of each model is lower than that for breast cancer and prostate cancer, but greater than that for lung cancer (Supporting Information S1: Figures 2 and 3). At a significance level of 0.05, the power is greater for Model 3 than Model 2 or Model 1 for OR <3 (Supporting Information S1: Figure 6).

### 3.5 Exome‐Wide Association Results Using Model 3

#### 3.5.1 Breast Cancer

Exome‐wide association analyses for breast cancer using this approach have been presented elsewhere (Wilcox et al. 2023). In brief, for PTVs, 30 genes were associated at *p* < 0.001. Of these, six genes reached exome‐wide significance (*p* < 2.5 × 10^–6^): the five previously known genes above plus *MAP3K1*. The results in Wilcox et al. were based on a meta‐analysis of UK Biobank and studies in the Breast Cancer Association Consortium, but the same six genes reached exome‐wide significance using UK Biobank alone. If the analyses had been based on Model 1 in UK Biobank alone, 23 genes would have met *p* < 0.001 compared to 27 using Model 3 (Supporting Information S1: Table 6). In UK Biobank, all the *p*‐values for the exome‐wide significant genes from Model 3 were at a factor of 1000 fold smaller than using Model 1, for example, for *MAP3K1* the *p*‐value for Model 3 was 3.2 × 10^–8^ compared to 8.3 × 10^–5^ in Model 1 (Supporting Information S1: Table 6).

We applied the same approach to three other common cancers for which family history data are available in UK Biobank: prostate, bowel, and lung.

#### 3.5.2 Prostate Cancer

For prostate cancer, 34 genes were associated at *p* < 0.001 (Supporting Information S1: Table 7, and Figures 7 and 8). Of these, three met exome‐wide significance; *BRCA2*, *CHEK2* and *ATM*. Associations at *p* < 1 × 10^–4^ were also identified for PTVs in *GEMIN2, OSGIN1, UBQLN4, C9orf50* and *C9orf152*. There was some evidence of association for *BAP1* (*p* = 0.0010) and *MSH4* (*p* = 0.0018). If the analyses had been based on Model 1, 26 genes would have met *p* < 0.001 compared with 34 using Model 3, and only two (*BRCA2* and *CHEK2*) would have met exome‐wide significance. The *p*‐values for the exome‐wide significant genes from Model 3 were all more significant than Model 1 and only two (*BRCA2* and *CHEK2*) would have met exome‐wide significance under Model 1.

#### 3.5.3 Lung Cancer

For lung cancer, 46 genes were associated at *p* < 0.001 (Supporting Information S1: Table 8 and Figures 9 and 10). Of these, none met exome‐wide significance, but associations at *p* < 1 × 10^–4^ were observed for *MON2, ASB6, ABCF2, PPP6R3, ARHGAP35, KCNH8* and *BIRC3*. However, we note that the number of case carriers for these genes was very low (≤4) and standard errors were large. Of known cancer susceptibility genes, some evidence of association was seen for *ATM* (*p* = 0.00012) and *BRCA2* (*p* = 0.0025). If the analyses had been based on Model 1, 56 genes would have met *p* < 0.001 compared with 46 using Model 3. However, the *p*‐values for the most significant genes from Model 3 were all more significant than Model 1, for example, for *ATM* the *p*‐value in Model 3 was 1.2 × 10^–4^ compared with 2.0 × 10^–3^ in Model 1 (Supporting Information S1: Table 8).

#### 3.5.4 Bowel Cancer

For bowel cancer, 42 genes were associated at *p* < 0.001 (Supporting Information S1: Table 9 and Figures 11 and 12). Of these, five met exome‐wide significance: the known susceptibility genes *MSH2, MSH6, MLH1* and *APC;* and *GAPDH*. The mismatch repair gene *PMS2* also showed evidence of association (*p* = 0.00020). Associations at *p* < 1 × 10^−4^ were also observed for *MT1G*, *FLCN, SMAD4* and *ATF3*. Among other cancer susceptibility genes, associations were seen for *ATM* (*p* = 0.0013), *BRCA1* (*p* = 0.0015), *BARD1* (*p* = 0.00021), *CHEK2* (*p* = 0.039), *RAD51D* (*p* = 0.0065), *MSH3* (*p* = 0.0025). If the analyses had been based on Model 1, 64 genes would have met *p* < 0.001 compared with 42 using Model 3. However, the *p*‐values for the exome‐wide significant genes were all more significant than Model 1, with no additional genome‐wide significant genes using Model 1, for example, for *GAPDH* the *p*‐value in model 3 was 9.3 × 10^–7^ compared to 1.9 × 10^–5^ in Model 1 (Supporting Information S1: Table 9).

## 4 Conclusions

Our analyses confirm that the inclusion of family history can improve the power of association studies, for both common variants (the focus of GWAS) and rare variants (the focus of WES studies). We show that for typical common variants, and for ‘moderate’ risk gene variants such as PTVs in *CHEK2* and *ATM*, a 1‐degree of freedom test (assigning a weight *k* = 1/2 to individuals with a family history) is more powerful than a 2‐degree of freedom test in which the effects of genotype on disease risk and family history are both estimated. This is consistent with the theoretical results that the 1‐degree of freedom testing with k = 1/2 should be most powerful in the limiting case of small effect sizes. The method can be implemented straightforwardly within any software providing logistic regression.

For variants with larger effect sizes, the 2df association test can be more powerful (or, equivalently, the 1df test can be made more powerful by assuming a larger *k*). This is logical: in the simplifying situation with a single relative, the relative risk associated with a positive family history $e^{\beta_{2}}$, should be approximately $\frac{1}{2}\left(e^{\beta_{1}}+1\right)$. When $\beta_{1}$ is large, $\beta_{2}{>\;\frac{1}{2}\beta }_{1}$, as is observed for *BRCA1*, *BRCA2* and *PALB2* for breast cancer. In practice, however, we expect that most novel variants will be associated with ORs < 2.5, corresponding to 0.5 < k < 0.6. In theory, the power would be improved by fixing *k* at its optimum value, but in practice, we do not know *k*, and *k* = 1/2 is a straightforward choice giving near optimum power. We also note that, in theory, it would be possible to fit the constrained model in which $e^{\beta_{2}}=\frac{1}{2}\left(e^{\beta_{1}}+1\right)$ rather than the simpler $\beta_{2}=\frac{1}{2}\beta_{1}$ constraint. However, this is a nonlinear model which is less suitable for genome‐wide analyses involving many genes or variants, and the model would still be approximate (e.g., dealing only approximately with cases with a family history).

It should be noted that fixing *k* = 0.5 will tend to overestimate $\beta_{1}$ if the effect size is large, as seen in the analysis the higher risk known genes. Thus, this is not an ideal approach for estimating risk. For risk estimation, a straightforward case–control or cohort analysis (ideally in a data set independent of the discovery data set) is recommended. However, the focus here is on genome‐wide discovery experiments, where the main interest is in the power to detect associations rather than effect size estimation for clinical application.

While the gain in power was seen for all cancers, it was particularly marked for lung cancer, reflecting the relatively large proportion of affected relatives (in turn related to the much higher risk in older individuals in early birth cohorts). Similarly, the approach is likely to be particularly informative for other late‐onset disease (e.g., dementia) where the number of affected relatives in older generations will be much larger than the number of disease cases in the cohort.

The gain in power by incorporating family history is illustrated by the exome‐wide association analyses for specific cancers. Using model 3 with *k* = 0.5, novel association genes at exome‐wide significance were identified for breast cancer, for example, *MAP3K1*, as reported elsewhere (Wilcox et al. 2023). For bowel cancer, we note *MSH2, MSH6, MLH1* and *PMS2* are MMR genes with known bowel cancer associations, while *APC* is a known susceptibility gene through its association with familial adenomatous polyposis. Biallelic *MSH3* variants have also been associated with adenomatous polyposis. To our knowledge the association for *GAPDH* is novel. Literature suggests *GAPDH* expression to be significantly upregulated in human colorectal carcinoma tissues compared to adjacent normal tissue (Tang et al. 2012). Genes associated with prostate cancer at exome‐wide significance include *BRCA2*, a known risk factor, as well as other breast‐cancer risk genes *CHEK2* and *ATM* for which previous evidence has been more equivocal. No significant associations were observed for *BRCA1* or *HOXB13*, previously identified risk genes (Ewing et al. 2012; Nyberg et al. 2020; Nyberg et al. 2019). However, the *BRCA1* association remains controversial, and there were only 12 case carriers in this data set, while the reported *HOXB13* association is specific to the p. Gly84Glu missense variant not included in these analyses. For lung cancer, no genes reached exome‐wide significance (using any model). The genes with more moderate evidence can, however, provide a basis for further targeted replication studies. There were more genes at *p* < 10^−4^ using Model 1 than Model 3 for lung and bowel cancer (in contrast to the theoretical expectations), however many of these genes had very low carrier counts and this may reflect a combination of chance and inaccuracy in the Type I error at very low frequencies.

While we have concentrated on the application of these methods to UK Biobank, the same approach could be fruitfully applied to other cohorts: for example, AllOfUs has family history for a wide range of cancers, as well as other diseases The “All of Us” Research Program 2019; Bick et al. 2024). Possible further developments to the model, in datasets where the information is available, would include incorporating more extensive family history information. Thus, in principle, it would be possible to extend the model to 2nd‐degree relatives (weighting by ¼). The method could also be extended to allow for multiple affected relatives (weighted by the degree of relationship). Including data on genotypes of relatives would further improve power.

In conclusion, these results demonstrate that including family history in burden regression models improves the power to identify cancer susceptibility genes. This is particularly relevant in the analysis of data from large cohort studies where the number of unaffected individuals outnumbers the number of affected individuals, and the number of unaffected individuals with a family history is significant. We demonstrated the power of this model for four cancer phenotypes, but the method could also be applied to non‐cancer phenotypes where family history information is available, such as Alzheimer's disease, major depressive disorder, and coronary artery disease in UK Biobank.

## Author Contributions

Douglas F. Easton supervised this work and directed the overall analysis. Naomi Wilcox performed the statistical analysis. Naomi Wilcox, Eugene J. Gardner, Jonathan P. Tyrer, Joe Dennis developed the bioinformatics and computational pipelines. Xin Yang and Joe Dennis acquired data and Xin Yang extracted cancer phenotypes. Naomi Wilcox and Douglas F. Easton drafted the manuscript. All authors reviewed and approved the paper.

## Conflicts of Interest

J.R.B.P. and E.J.G. are employees of Insmed Innovation UK and hold stock/stock options in Insmed Inc. J.R.B.P. also receives research funding from GSK and engages in paid consultancy for WW International Inc.

## Supporting information

Supporting information.

## Data Availability

We are currently in the process of submitting results to https://www.ebi.ac.uk/gwas/.
